# Basal adenosine modulates the functional properties of AMPA receptors in mouse hippocampal neurons through the activation of A_1_R A_2A_R and A_3_R

**DOI:** 10.3389/fncel.2015.00409

**Published:** 2015-10-12

**Authors:** Silvia Di Angelantonio, Cristina Bertollini, Sonia Piccinin, Maria Rosito, Flavia Trettel, Francesca Pagani, Cristina Limatola, Davide Ragozzino

**Affiliations:** ^1^Istituto Pasteur-Fondazione Cenci Bolognetti and Dipartimento di Fisiologia e Farmacologia, Sapienza Università di RomaRoma, Italy; ^2^Center for Life Nanoscience, Istituto Italiano di TecnologiaRome, Italy; ^3^Neuromed, Istituto di Ricovero e Cura a Carattere ScientificoPozzilli, Italy

**Keywords:** adenosine receptors, AMPA receptors, hippocampal neurons, neuronal modulation, A_3_R

## Abstract

Adenosine is a widespread neuromodulator within the CNS and its extracellular level is increased during hypoxia or intense synaptic activity, modulating pre- and postsynaptic sites. We studied the neuromodulatory action of adenosine on glutamatergic currents in the hippocampus, showing that activation of multiple adenosine receptors (ARs) by basal adenosine impacts postsynaptic site. Specifically, the stimulation of both A_1_R and A_3_R reduces AMPA currents, while A_2A_R has an opposite potentiating effect. The effect of ARs stimulation on glutamatergic currents in hippocampal cultures was investigated using pharmacological and genetic approaches. A_3_R inhibition by MRS1523 increased GluR1-Ser845 phosphorylation and potentiated AMPA current amplitude, increasing the apparent affinity for the agonist. A similar effect was observed blocking A_1_R with DPCPX or by genetic deletion of either A_3_R or A_1_R. Conversely, impairment of A_2A_R reduced AMPA currents, and decreased agonist sensitivity. Consistently, in hippocampal slices, ARs activation by AR agonist NECA modulated glutamatergic current amplitude evoked by AMPA application or afferent fiber stimulation. Opposite effects of AR subtypes stimulation are likely associated to changes in GluR1 phosphorylation and represent a novel mechanism of physiological modulation of glutamatergic transmission by adenosine, likely acting in normal conditions in the brain, depending on the level of extracellular adenosine and the distribution of AR subtypes.

## Introduction

Purinergic signaling is emerging as one important mechanism of integrating functional activity between neurons, glial and vascular cells in the brain, through the action of both ATP and adenosine signaling (Burnstock et al., 2011). Adenosine is a cellular metabolite formed by the breakdown of adenine nucleotides and is physiologically present at nanomolar to micromolar concentrations inside and outside the cells (Fredholm, 2007). It represents an endogenous modulator of brain functions, fine-tuning glial function (Boison, 2007), blood flow (Kusano et al., 2010), but also inhibitory and excitatory synaptic transmission (Ribeiro and Sebastião, 2010); moreover, in the nervous system, the level of extracellular adenosine rises following brain damage, which occurs after stroke, ischemia, and epileptic seizures (Ribeiro et al., 2003).

Basal extracellular adenosine concentration depends on the activity of both membrane nucleoside transporters (Parkinson et al., 2011; Diógenes et al., 2014), and extracellular ecto-nucleotidases (Zimmermann et al., 2012). It has been reported that the synaptic concentration of adenosine, tightly controlled by astrocytes and neurons, is usually higher than the intracellular one, and tonically modulates synaptic activity acting on adenosine receptors (ARs) A_1_R and A_2A_R to regulate pre or post synaptic functions (Sebastião and Ribeiro, 2014). The best characterized effect of adenosine is the presynaptic modulation of glutamatergic transmission (Dunwiddie and Fredholm, 1984): presynaptic activation of A_1_R reduces glutamate release (Sebastião et al., 1990), while activation of A_2A_R increases glutamatergic transmission (Sebastião and Ribeiro, 1992; Cunha et al., 1994; Lopes et al., 2002).

It is also known that adenosine may modulate synaptic transmission also acting at the postsynaptic level (Haas and Greene, 1984; Sebastião and Ribeiro, 2014), regulating the activity of neurotransmitter receptors. Specifically, A_1_R activation inhibits AMPA and NMDA induced currents in the hippocampus (de Mendonça et al., 1995; Piccinin et al., 2010; Dias et al., 2012), while A_2A_R stimulation increases the amplitude of AMPA-evoked and miniature postsynaptic currents enhancing GluR1 phosphorylation at Ser845 (Dias et al., 2012). By contrast, few studies have focused on the role of A_3_ receptors (A_3_R) in synaptic modulation, although these receptors have been identified in neurons (Lopes et al., 2003; Rebola et al., 2005), astrocytes and microglial cells (Boison et al., 2010) and have a potential impact in glutamatergic modulation and synaptic plasticity (Costenla et al., 2001; Maggi et al., 2009; Piccinin et al., 2010), in addition to their involvement in pain modulation (Ford et al., 2015) and neuroprotection (Chen et al., 2006; Pugliese et al., 2007; Dennis et al., 2011; Rosito et al., 2012, 2014).

In this study we demonstrate the ability of A_3_R to negatively modulate postsynaptic AMPA receptors through the reduction of GluR1 phosphorylation level. Moreover, this effect is synergistic with the A_1_R mediated reduction of AMPA currents, and counteracts the potentiating effect of A_2A_R activation. Importantly, we show that adenosine modulation is tonically active. Hence, the concomitant activity of A_1_R, A_2A_R, and A_3_R tightly tunes glutamatergic synapses, setting the properties of AMPA receptors functioning in basal conditions.

## Materials and Methods

### Animals

Procedures using laboratory animals were in accordance with the Italian and European guidelines and were approved by the Italian Ministry of Health in accordance with the guidelines on the ethical use of animals from the European Community Council Directive of 22 September 2010 (2010/63/EU). All efforts were made to minimize the number of animals used and their suffering.

### Primary Hippocampal Neuronal Cultures

Hippocampal neuronal cultures were prepared as reported in Di Angelantonio et al. (2014), from newborn (P0-P1) C57BL/6 and KO mice of either sex (Charles River Laboratory). In brief, after careful dissection from diencephalic structures, the meninges were removed and the hippocampi were chopped and digested in 1.25 mg/ml trypsin for 20 min at 37°C. Cells were mechanically dissociated and plated at a density of 105 in poly-L-lysine coated glass coverslip (12 mm diameter) in serum-free Neurobasal medium, supplemented with B27 plus 2 mM L-glutamine and 100 μg/ml gentamicin (neuronal culture medium). Then, cells were kept at 37°C in 5% CO_2_ for 10–13 days with medium replacement (1:1 ratio) three times per week. With this method we obtained cultures composed by 60–70% neurons, 30–35% astrocytes, and 4–5% microglia, as determined with β-tubulin III, glial fibrillary acidic protein (GFAP), and isolectin IB4 staining (Lauro et al., 2008). The same procedure was followed to prepare rat hippocampal culture used for some immunoblot experiments.

### Glial Primary Cultures

Primary cortical glial cells were prepared from P0–P2 mice. Cerebral cortices were chopped and digested in 30 U/ml papain for 40 min at 37°C and gently triturated. The dissociated cells were washed, suspended in Dulbecco’s Modified Eagle Medium (DMEM, GIBCO) with Glutamax with 10% FBS (Invitrogen) and plated at a density of 9–10 × 105 in 175 cm^2^ cell culture flasks. At confluence (10–14 days *in vitro*, DIV), glial cells were shaken for 2 h at 37°C, to detach and remove microglial cells. These procedures gave almost pure astrocytes cell population (4–6% of microglia contamination), as verified by staining with GFAP and isolectin IB4 (Rosito et al., 2012).

### Slice Preparation

Hippocampal slices were routinely prepared from 14- to 20-days old C57BL/6 mice (WT), A_1_R knockout mice (A_1_R KO; Johansson et al., 2001), and the A_3_R knockout mice (A_3_R KO; Salvatore et al., 2000). The latter two groups of mice were backcrossed at least 10 times on a C57BL/6 background. Mice were decapitated under halothane anesthesia, and whole brains were rapidly removed and incubated in chilled artificial CSF (ACSF) for 15 min. Transverse hippocampal slices (250 μm) were cut at 4°C, using a Vibratome (DSK, Dosaka EM, Kyoto, Japan). Before use, slices were maintained for at least 1 h at room temperature (22–25°C) in oxygenated (95% O_2_, 5% CO_2_) ACSF, containing the following (in mM): 125 NaCl, 2.5 KCl, 1.25 NaH_2_PO_4_, 26 NaHCO_3_, 2 CaCl_2_, 1 MgCl_2_, and 10 glucose, pH 7.35. All recordings were performed at room temperature on slices submerged in ACSF in the recording chamber. The ACSF was perfused at a rate of 1 ml/min.

### Patch-clamp Recordings

Patch-clamp recordings were obtained using glass electrodes (3–5 MΩ) filled with the following intracellular solution (in mM): 140 KCl, 2 MgCl_2_, 10 HEPES, 2 MgATP, 0.5 EGTA; pH 7.3, with KOH. During experiments, cultured neurons were continuously superfused with normal extracellular solution (NES) containing (in mM): 140 NaCl, 2.5 KCl, 2 CaCl_2_, 2 MgCl_2_, 10 HEPES-NaOH, and 10 glucose (pH 7.3), added with tetrodotoxin (0.2 μM), using a gravity driven perfusion system, consisting of independent tubes for standard and agonist-containing solutions, connected to a fast exchanger system used to delivery all agonists (RSC-100; Bio-Logic). To activate glutamate receptors in hippocampal neurons in acute slices, AMPA was delivered together with cyclothiazide (25 μM; Tocris Cookson) by pressure application (5–15 psi, 10–100 ms; Picospritzer II, General Valve, Fairfield, NJ, USA) from glass micropipettes positioned above the slice over the soma of the recorded neuron. All other agonists were applied to hippocampal slices by bath perfusion.

In all recordings, AMPA was delivered together with cyclothiazide, to avoid fast receptor desensitization (Fucile et al., 2006) that could mask adenosine mediated AMPA receptor modulation. Cyclothiazide was always delivered in co-application with the agonist. Agonist concentration–current curves were constructed applying to each cell two or more different concentrations of agonist (10 nM–100 μM) at 60–120 s intervals and normalizing the current response to the plateau value, tested in all cells. Averaged data were best-fitted using Origin 7 (OriginLabCorp., Northampton, MA, USA) to the Hill equation:

$${\text{I}}_{\text{norm}}\;\text{=}\;\text{1/}(\text{1}\;\text{+}\;{\left({\text{EC}}_{\text{50}}\right)}^{\text{nH}}\text{/}{\left[\text{Agonist}\right]}^{\text{nH}})$$

Where I_norm_ is the normalized current response, EC_50_ is the agonist concentration yielding half-maximal current response and nH is the Hill coefficient.

Membrane currents, recorded with a patch-clamp amplifier (Axopatch 200B; Molecular Devices, Foster city, CA, USA), were filtered at 2 kHz, digitized (10 kHz), and acquired with Clampex 10 software (Molecular Devices). The stability of the patch was checked by repetitively monitoring the input and series resistance during the experiment, and recordings were discarded when any of these parameters changed by >10%.

All recordings were performed at 24–25°C, and cells were patch clamped at -70 mV.

### Cell Stimulation and Western Blot Analysis

Ten DIV hippocampal cultures were incubated in Locke’s buffer (in mM): 154 NaCl, 5.6 KCl, 3.6 NaHCO_3_, 2.3 CaCl_2_, 1 MgCl_2_, 5.6 glucose, buffered with 5 HEPES (pH 7.4) for 2 h and stimulated for 30 min with MRS1523 (100 nM).

Cells were then washed with PBS, scraped and lysed (Tris-HCl 50 mm pH 7.5, NaCl 150 mm, EGTA 1 mm, EDTA 1 mm, 1%, Triton X-100, 0.1% SDS, phosphatase and protease inhibitor mixture). Protein concentration was determined by BCA assay (Pierce), and the same amounts of proteins (30 μg) were separated on 10% SDS-PAGE and analyzed by Western immunoblot using with antibodies specific for phospho-GluR1 Ser845 (Upstate Biotechnology, Lake Placid, NY, USA), and actin (Sigma). Immunoreactivity was detected by chemiluminescence (Immun-Star WesternC Kit; Bio-Rad). Specific bands on chemiluminescence films were quantified by densitometry with Sigma Gel Software (Jandel Scientific, Erkrath, Germany).

### Drugs

SCH5821 (stock solution 10 mM in DMSO), DPCPX (stock solution 5 mM in DMSO), 2-Cl-IBMECA (stock solution 10 mM in DMSO), Bicuculline methochloride (stock solution 100 mM), AMPA (stock solution 100 mM), cyclothiazide (stock solution 50 mM in DMSO) and Tetrodotoxin citrate (stock solution 1 mM) were purchased from Tocris Bioscience, Bristol, UK. MRS1523 (stock solution 100 mM in DMSO) and all other drugs used were purchased from Sigma–Aldrich, Milan, Italy. Where not indicated, all drugs were dissolved in water.

### Drug Application

During electrophysiological measurements, neurons were continuously superfused with NES, using a gravity driven perfusion system, consisting of independent tubes for standard and agonist-containing solutions, connected to a fast exchanger system (RSC-100; Bio-Logic) positioned 50–100 μm from the cell. Antagonists were usually acutely applied through parallel tubes of the same perfusion system or were pre-incubated for 1 h (37°C) and then continuously applied during the experiments.

### Data Analysis

Data, analyzed oﬄine, are presented as mean ± SEM; we used the QuantityOne (Biorad) program for the densitometric analysis of all immunoblots. Origin 7 (Origin software; Microcal Software, Northampton, MA) and Sigmaplot 11 (Jandel scientific) software were used for statistical analysis. Paired and unpaired *t*-test and one-way ANOVA were used for parametrical data, as indicated; Holm–Sidak test was used as *post hoc* test; Mann and Withney test for non-parametrical data.

## Results

### Modulation of AMPA Receptors by Basal Adenosine

**FIGURE 1:**
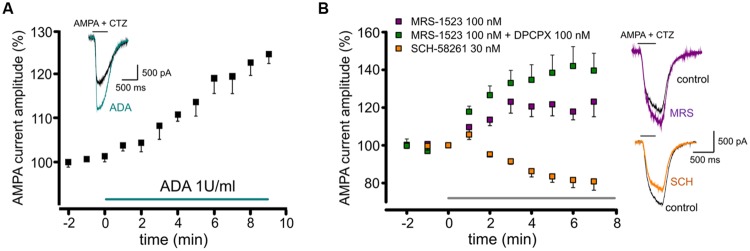
**Tonic activation of adenosine receptors modulates AMPA currents in mouse hippocampal neurons. (A)** time course and sample traces of AMPA current (AMPA 10 μM, CTZ 25 μM) potentiation induced by acute treatment with Adenosine deaminase (ADA, 1 U/ml, *n* = 5; *p* < 0.05) in cultured hippocampal neurons. **(B)** Left, time course of AMPA current modulation by acute treatment with adenosine receptors antagonists blocking specifically: A_3_R (MRS1523, 100 nM, *n* = 5, purple), A_3_R + A_1_R (MRS1523 + DPCPX, *n* = 5, green) or A_2A_R (SCH58261 30 nM, *n* = 5, orange). Application of all drugs starts at *t* = 0, gray bar. Right, sample traces of AMPA currents in control (black) and in the presence of MRS1523 (purple) or SCH58261 (orange).

It is well known that adenosine is present in brain extracellular space, as well as in neural preparations, such as brain slices or neuronal cultures. When we removed basal adenosine in hippocampal cultures with adenosine deaminase (ADA; 1 U/ml; 10 min), we observed a slow increase in the amplitude of currents evoked by AMPA application (10 μM; plus cyclothiazide, CTZ, 25 μM; to 120 ± 4%, *n* = 5, *p* < 0.05; **Figure 1A**). This result suggests that adenosine present in the extracellular medium tonically depresses glutamatergic transmission by modulating the amplitude of AMPA currents.

To unveil the involvement of AR subtypes in AMPA current modulation, cultures were treated with specific ARs antagonists, in order to establish the possible contribution of A_1_R, A_2A_R, and A_3_R.

When control cultures were superfused with the A_3_R specific antagonist MRS1523 (100 nM, 8 min), we observed a significant increase in the amplitude of AMPA currents (to 123.1 ± 7.9%, *n* = 5; *p* < 0.05; **Figure 1B**). A similar effect was obtained treating cultures with the A_1_R specific antagonist DPCPX (100 nM), causing current amplitude increase to 123.8 ± 13.5% (*n* = 10, *p* < 0.05; not shown). Interestingly, when both A_3_R and A_1_R were blocked the current increase was significantly higher, suggesting an additive effect of the two antagonist (*n* = 5, *p* < 0.05 vs. MRS1523 or DPCPX alone, one way ANOVA, Holm–Sidak, **Figure 1B**).

Conversely, the application of the specific A_2A_R antagonist SCH58261 (30 nM; 8 min) caused a decrease in current amplitude (to 80.8 ± 4.6%; *n* = 5, *p* < 0.05; **Figure 1B**).

In addition, prolonged treatment of hippocampal cultures with specific ARs antagonists (30 min preincubation and during recordings) significantly affected the AMPA current response amplitude (10 μM plus CTZ, 25 μM; **Table 1**); the mean current amplitude was in fact significantly enhanced by MRS1523 or DPCPX treatment, while it was reduced by SCH58261 (**Table 1**).

**Table 1:** Dose response parameters of AMPA response.

	Peak amplitude (pA) (*n*)	EC_50_ (μM) (*n*)	n_H_
WT	678 ± 64 (53)	5.4 ± 0.7 (32)	1.6 ± 0.1
MRS1523	1740 ± 520 (13)^∗∗^	1.3 ± 0.3 (11)^∗∗^	1.9 ± 0.3
DPCPX	1420 ± 180 (12)^∗∗^	1.6 ± 0.2 (12)^∗^	2.1 ± 0.4
SCH58261	504 ± 54 (9)^∗^	8.6 ± 0.5 (9)^∗∗^	1.4 ± 0.3
A_3_R KO	1020 ± 93 (33)^∗∗^	1.2 ± 0.3 (11)^∗∗^	1.8 ± 0.2
A_1_R KO	888 ± 130 (20)^∗^	1.9 ± 0.3 (8)^∗^	1.7 ± 0.3
A_2A_R KO	754 ± 210 (13)	10.5 ± 0.6 (12)^∗∗^	1.5 ± 0.1

*Data represent means ± SEM (number of cells) of: Column 2, mean Peak amplitude response to a test dose of agonist (AMPA 10 μM); Column 3, 4 fit parameters for dose-response curve fitted by Hill equation. ^∗^*p* < 0.05 and ^∗∗^*p* < 0.01, significantly different from control value in WT untreated neurons, as specified in Column 1.*

These data indicate that in hippocampal cultures, ARs are tonically active due to the presence of adenosine in the extracellular medium, and that this has a modulatory effect on glutamatergic currents. The stimulation of A_3_R or A_1_R subtypes causes a tonic depression of AMPA receptors, while A_2A_R activation potentiates them; under basal condition, the inhibitory tone prevails, suggesting a predominance of A_1_R, A_3_R-mediated AMPAR modulation.

### AMPA Receptors Affinity is Modulated by the Selective Activation of ARs

To understand how ARs activation could affect the AMPA receptor function, we measured the AMPA concentration–current response curve in hippocampal neurons, both in control conditions or interfering with basal ARs activation. In particular, to unveil the contribution of single AR subtypes to GluRs modulation, we selectively abolished specific ARs activity either pharmacologically or genetically, using hippocampal cultures derived from A_1_R, A_2A_R od A_3_R KO mice.

**FIGURE 2:**
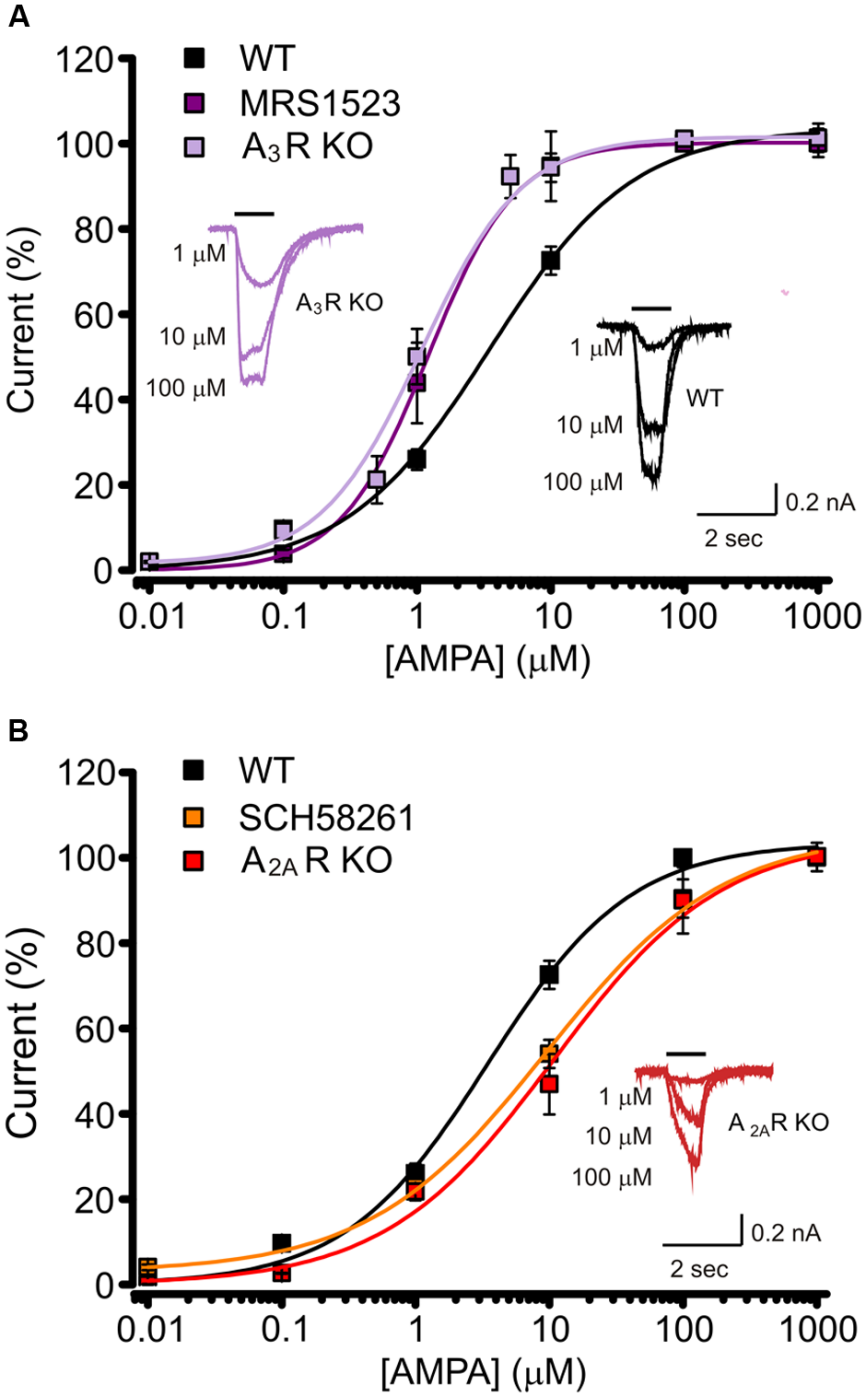
**Basal activation of AR subtypes changes the apparent affinity for AMPA. (A)** AMPA dose response curves (+ CTZ 25 μM) obtained in cultured hippocampal neurons in control (black, *n* = 32), in the presence of A_3_R antagonist MRS1523 (100 nM, *n* = 11, purple) and in neurons from A_3_R KO mice (*n* = 11, magenta). Inserts: sample traces representing current responses to 1, 10, and 100 μM AMPA (+ CTZ 25 μM) in WT and A_3_R KO hippocampal neurons. **(B)** AMPA dose response curves (+ CTZ 25 μM) obtained in cultured hippocampal neurons in the presence of A_2A_R antagonist SCH58261 (30 nM, *n* = 9, orange) and in neurons from A_2A_R KO mice (*n* = 12; red; control condition in black). Inserts: representative current responses to 1, 10, and 100 μM AMPA (+ CTZ 25 μM) in A_2A_R KO hippocampal neurons.

When WT cells were treated with MRS1523 (100 nM), to block A_3_R, we observed a leftward shift in the AMPA concentration–response curve (**Figure 2A**), corresponding to an increase in the agonist apparent affinity (**Figure 2A**; **Table 1**, *n* = 11) with a EC_50_ value significantly different from the one observed in control conditions (**Table 1**, *n* = 32, *p* < 0.01), suggesting that basal A_3_R activation reduces GluR sensitivity for the agonist. Consistently, we observed an increase in the agonist affinity in hippocampal cultures derived from A_3_R KO mice (*n* = 11; **Figure 2A**; **Table 1**, *p* < 0.01).

Analogously, both the pharmacological inhibition by DPCPX (10 nM; *n* = 12) and the genetic deletion of A_1_R (hippocampal cultures derived from A_1_R KO mice), caused an increase in AMPA potency (**Table 1**, *n* = 8; *p* < 0.05).

Conversely, when A_2A_R was inhibited by SCH58261 or deleted in A_2A_R KO cultures, the apparent affinity for AMPA was decreased (*n* = 9, *p* < 0.05 and *n* = 12, *p* < 0.01 respectively; **Figure 2B**; **Table 1**).

Altogether, these data indicate that GluRs functional properties are finely tuned by activation of different AR subtypes by basal adenosine. The sensitivity of GluRs is modulated in opposite directions by activation of ARs and in particular, A_3_R or A_1_R decrease agonist affinity, while it is increased by A_2A_R.

### Neuronal A_3_R Regulates AMPA Receptors Function Through Modulation of GluR1-Ser845 Phosphorylation

**FIGURE 3:**
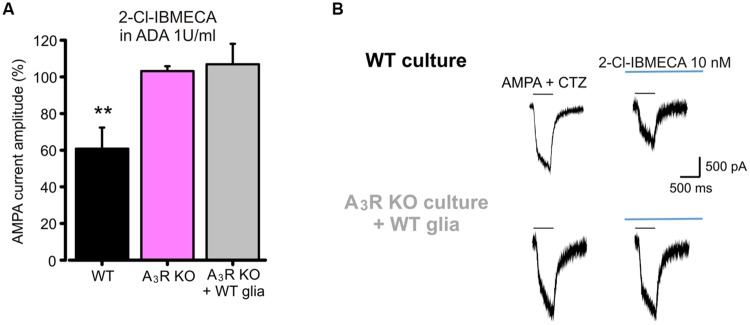
**AMPARs function modulation requires neuronal A_3_R activation. (A)** Reduction of AMPA current amplitude induced by the application of A_3_R agonist 2-Cl-IBMECA in cultured hippocampal neurons treated with Adenosine deaminase (1 U/ml, 1 h preincubation + perfusion); bar chart representing the effect observed in WT cultures (black; *n* = 12); or A_3_R KO cultures (magenta; *n* = 13); gray column represents the effect of 2-Cl-IBMECA in A_3_R KO cultures in the presence of WT glial cells (*n* = 12). **(B)** Sample traces showing the effect of 2-Cl-IBMECA on AMPA currents in hippocampal neurons from WT (top) and A_3_R KO (bottom) mice, co-cultured with WT glial cells. ^∗∗^*p* < 0.01.

To investigate the cellular and molecular determinants underlying the A_3_R mediated modulation of AMPA currents, we performed experiments using the specific A_3_R agonist 2-Cl-IBMECA. To remove basal activation of ARs, control hippocampal cultures were treated with ADA (1 U/ml, 1 h pre-application and during the experiment) and current amplitude was monitored during the application of the specific A_3_R agonist 2-Cl-IBMECA. Consistently with the above reported results with A_3_R antagonist, the application of 2-Cl-IBMECA (10 nM, 10 min) reduced the AMPA current amplitude (to 60 ± 11%, *n* = 12, *p* < 0.01; **Figure 3A**).

As expected, in cultures prepared from A_3_R KO mice, 2-Cl-IBMECA failed to reduce AMPA current (103 ± 3%; *n* = 13; *p* = 0.67; **Figure 3A**), confirming that the observed effect was ascribable to specific A_3_R stimulation. To evaluate the possible contribution of glial A_3_R in the AMPA current modulation, we explored if glial A_3_R stimulation could rescue AMPA current modulation. To this purpose, A_3_R KO hippocampal cultures were seeded on a feeding layer of either A_3_R KO or WT glial cells: 2-Cl-IBMECA was ineffective both in A_3_R KO cultures seeded on WT glia (*n* = 14, *p* = 0.84; **Figures 3A,B**) and co-cultured with A_3_R KO glia (*n* = 12, *p* = 0.91), strongly indicating that 2-Cl-IBMECA acts specifically on neuronal A_3_R to modulate AMPA function.

**FIGURE 4:**
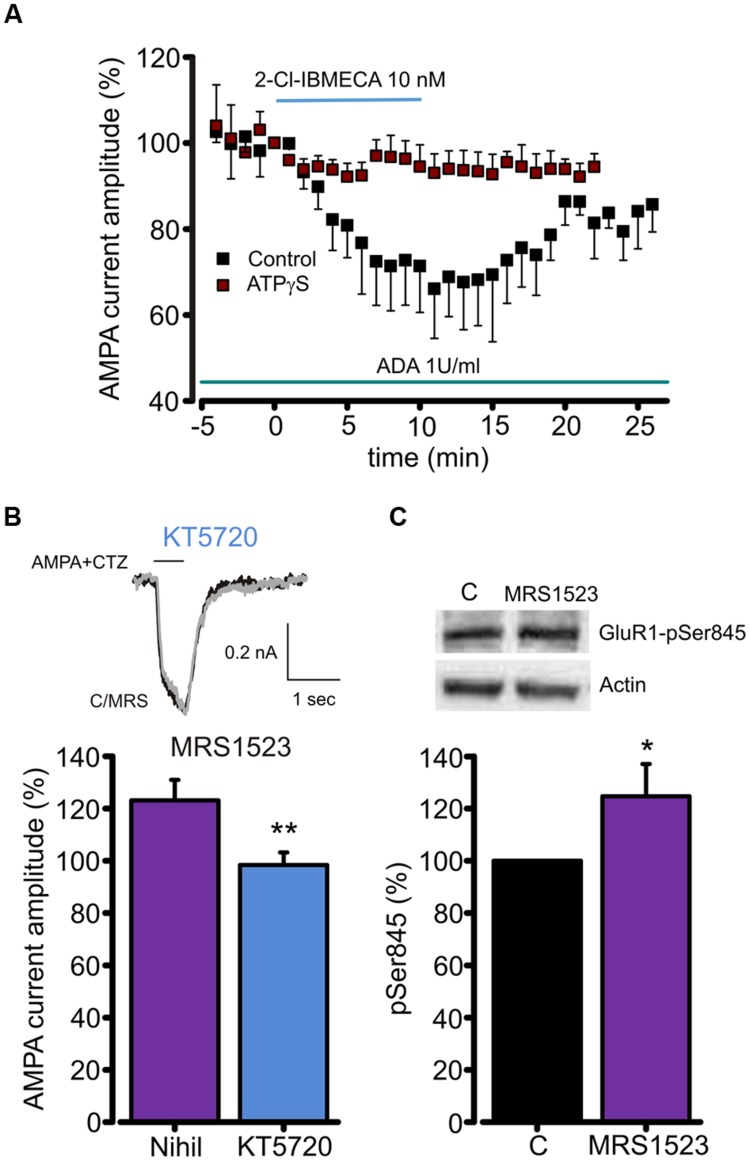
**A_3_R dependent AMPARs modulation requires GluR1 dephosphorylation. (A)** Time course of the effect of the A_3_R agonist 2-Cl-IBMECA on AMPA current amplitude in the continuous presence of ADA (1 U/ml, 1 h preincubation) in control condition (black, *n* = 12) and after substitution of intracellular ATP with the thiophosphorylating agent ATPγS (*n* = 6). **(B)** Bar chart and sample traces displaying mean AMPA current potentiation induced by A_3_R antagonist MRS1523 in control and in the presence of PKA blocker KT5720 (*n* = 6). **(C)** Representative immunoblot and quantitative western blot analysis of the effect of MRS1523 on the level of basal Glur1 Ser845 phosphorylation (*n* = 13). ^∗^*p* < 0.05, ^∗∗^*p* < 0.01.

Since A_3_R stimulation reduces cAMP accumulation (Zhou et al., 1992), and PKA is responsible for GluR1 phosphorylation at Ser845 (Banke et al., 2000), we studied the contribution of A_3_R in AMPAR phosphorylation to understand the intracellular pathway leading to the AMPA current modulation. To address this issue, ATP was substituted in the intracellular solution by ATPγS, in order to promote irreversible substrate thio-phosphorylation (Eckstein, 1985) and thus to prevent the AMPAR dephosphorylation. As reported in **Figure 4A**, in this experimental condition 2-Cl-IBMECA (10 nM, ADA 1U/ml, *n* = 6, *p* < 0.05) failed to reduce the AMPA current amplitude. Consistently, MRS1523 (100 nM) application failed to potentiate AMPA-mediated currents when neuronal PKA was inhibited by intracellular perfusion with KT5720 (**Figure 4B**; *n* = 6; *p* = 0.83).

To test whether A_3_R modulation of AMPA currents could be associated to changes in GluR1 phosphorylation level, we exposed hippocampal cultures to MRS1523 (100 nM, 30 min) and evaluated the level of GluR1 phosphorylation at Ser845. Quantitative western blot analysis showed that, in MRS1523 treated cultures, the Ser845 phosphorylation increased to 125.6 ± 11.8% (*n* = 13, *p* < 0.03; **Figure 4C**), indicating that tonic A_3_R activity reduces GluR1 phosphorylation. Altogether, these results indicate that the A_3_R activation modulates GluR functions through receptor dephosphorylation.

### Modulation of Postsynaptic AMPA Receptors in Acute Hippocampal Slices by A_1_R, A_2A_R and A_3_R Activation

**FIGURE 5:**
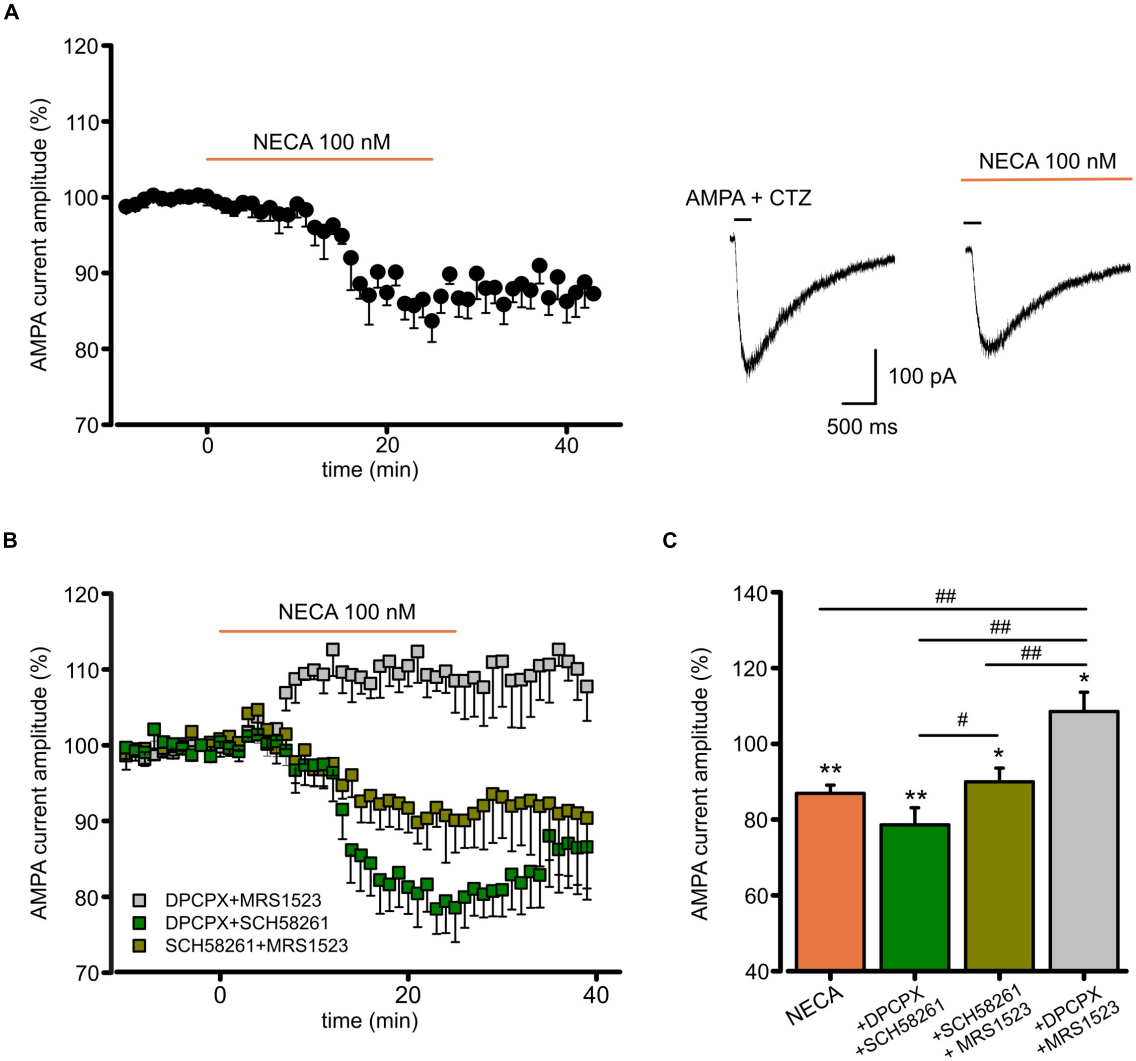
***N*-ethyl 1-5′ carboxamido adenosine (NECA) modulates postsynaptic AMPA receptors through multiple ARs. (A)** Time course and representative traces of AMPA (100 μM plus CTZ 25 μM) current amplitude reduction induced by NECA (*t* = 25 min) (100 nM) in hippocampal CA1 neurons (*n* = 6; *p* < 0.01). **(B,C)** Time course and bar chart (at *t* = 25 min) of the effect of NECA in the presence of combination of ARs antagonists: DPCPX (100 nM) plus MRS1523 (100 nM) (gray; *n* = 6; *p* < 0.01); SCH58261 (30 nM) plus MRS1523 (dark green; *n* = 6; *p* < 0.05); DPCPX plus SCH58261 (green; *n* = 5; *p* < 0.01). ^∗^*p* < 0.05, ^∗∗^*p* < 0.01, ^#^*p* < 0.05, ^##^*p* < 0.01.

To disclose the postsynaptic modulation of glutamatergic currents by ARs activation in hippocampal slices, we recorded the effect of the high affinity AR agonist *N*-ethyl 1-5′ carboxamido adenosine (NECA) application on currents evoked by repetitive pressure application of AMPA (100 μM, plus CTZ 25μM) on CA1 pyramidal neurons. Prolonged NECA application (100 nM; 25 min) caused a reduction in the AMPA current amplitude (to 86.9 ± 2.2%, *p* < 0.01 paired *t*-test; *n* = 6; **Figure 5A**), which did not recover within 20 min after drug washout (**Figure 5A**). We used combinations of specific ARs antagonist to disclose the contribution of single receptor subtypes (**Figure 5B**). In the presence of DPCPX (100 nM) plus SCH58261 (30 nM), allowing only the activation of A_3_R, NECA caused a reduction of the AMPA current amplitude (to 78.6 ± 4.5%; *n* = 5; *p* < 0.01, paired *t*-test), followed by a slow recovery after drug withdrawal. Smaller depression (to 90.0 ± 3.6%; *n* = 6, *p* < 0.05, paired *t*-test) was observed when NECA was applied in the presence of SCH58261 plus MRS1523 (100 nM), allowing only the activation of A_1_R (*p* < 0.05 respect to NECA in the presence of DPCPX plus SCH58261; One way ANOVA, Holm–Sidak, **Figure 5C**). Conversely, in the presence of DPCPX plus MRS1523, NECA caused AMPA current potentiation, due to A_2A_R stimulation (to 108.5 ± 5.1%, *n* = 6, *p* < 0.05, paired *t*-test). All together, these results indicate that also in hippocampal slices, activation of A_1_R, A_2A_R and A_3_R modulates AMPA receptors.

**FIGURE 6:**
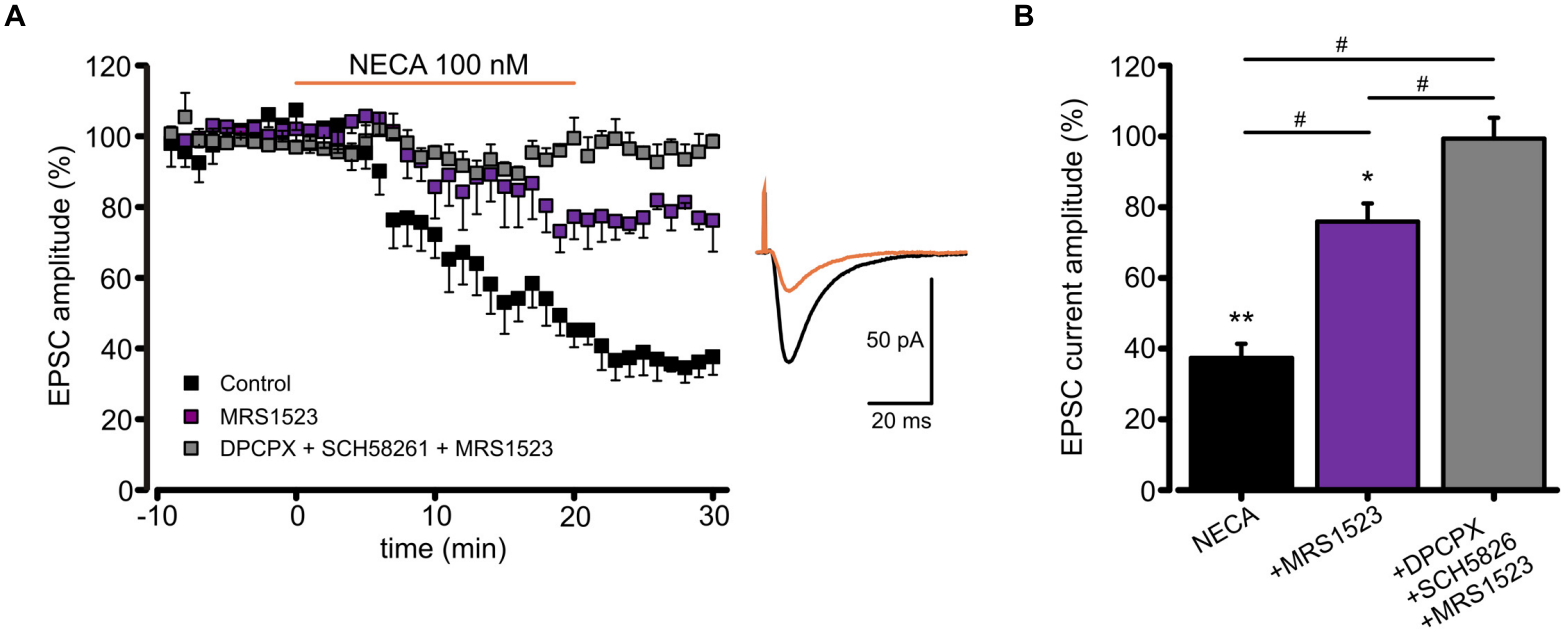
**Modulation of EPSC amplitude by NECA depends on A_3_R. (A)** *Left*, time course of EPSC depression induced by adenosine receptor agonist NECA (100 nM) in hippocampal CA1 neurons in from acute slices in control conditions *(black; n* = 7; *p* < 0.05 at *t* = 25 min), in the presence of A_3_R antagonist MRS1523 (100 nM) (purple; *n* = 7; *p* < 0.05 respect to control and NECA alone) and in the presence of DPCPX + SCH58261 + MRS1523 (gray, *n* = 7). *Right*, sample EPCSs recorded in a CA1 pyramidal neuron in control and following NECA application. **(B)** Bar chart showing the effect of NECA (100 nM) alone (black), in the presence of the A_3_R antagonist (MRS1523 100 nM, purple) and in the presence of all three blockers (DPCPX + SCH58261+MRS1523 gray, *n* = 7). ^∗^*p* < 0.05, ^∗∗^*p* < 0.01, ^#^*p* < 0.05.

To verify whether ARs modulation of AMPA receptors might affect glutamatergic synaptic transmission, we recorded, in CA1 pyramidal neurons, the excitatory postsynaptic currents (EPSCs) evoked by Schaffer collaterals stimulation. In this condition, we observed that basal adenosine, present in the slice preparation, modulates glutamatergic synaptic currents being EPSC amplitude increased by acute ADA application (Supplementary Figure S1A). Moreover, as revealed using A_1_RKO and A_3_RKO mice, the application of high dose of adenosine induced an EPSC depression depending on both A_1_R and A_3_R (Supplementary Figures S1B,C). In addition, NECA application caused a reduction of EPSCs amplitude (*n* = 7; *p* < 0.05; **Figure 6A**), without affecting the paired pulse ratio (not shown), supporting a postsynaptic action of NECA that largely depends on the A_3_R activation. Indeed, NECA-mediated EPSCs depression was significantly smaller when A_3_R was blocked (MRS1523 100 nM, *n* = 4, *p* < 0.05 respect to NECA alone, one way ANOVA, Holm–Sidak; **Figure 6B**), and it was abolished when A_1_R and A_2A_R blockers (DPCPX 100 nM, SCH58261 30 nM) were simultaneously added (*n* = 7, *p* < 0.01 respect to NECA alone, one way ANOVA, Holm–Sidak; **Figure 6B**).

These data confirm that the A_3_R activation may modulate glutamatergic synaptic transmission by a postsynaptic mechanism affecting AMPA receptor functions.

## Discussion

We report here that adenosine present in the extracellular milieu may tonically regulate the function of AMPA receptors acting on A_1_R, A_2A_R, and A_3_R. Our data show that adenosine exerts multiple effects on glutamatergic transmission, modulating in opposite direction the apparent affinity of AMPA receptors, depending on AR subtypes. Our study is particularly focused on the effects of A_3_R stimulation, highlighting the role of this AR subtype in the modulation of glutamatergic transmission.

We propose that the A_3_R modulation of AMPA receptors is based on the alteration of the phosphorylation balance, at GluR1-Ser845, leading to changes in the apparent agonist affinity of the receptor. It is well known that phosphorylation affects the properties of the glutamate receptors (Banke et al., 2000) shifting the sensitivity to the transmitter/agonist (Lax et al., 2002); similar mechanisms could be involved in adenosine-based forms of synaptic modulation and plasticity (Ragozzino et al., 2006; Lauro et al., 2008; Piccinin et al., 2010; Scianni et al., 2013). Our data show that the A_3_R stimulation causes a depression of the AMPA currents, which is likely associated with receptor dephosphorylation, since it is absent in the presence of ATPγS which promotes irreversible thio-phosphorylation of substrates (Eckstein, 1985). Consistently, the treatment with the specific A_3_R blocker MRS1523 (i) causes the increase in the AMPA current amplitude, as confirmed in experiments on A_3_R KO cultures; (ii) is associated to the increase in the level of GluR1-Ser845 phosphorylation and (iii) is blocked by the PKA antagonist KT5720. Indeed, adenosine is known to act through intracellular pathways involving PKA (Fredholm et al., 2001a), potentially interfering with the phosphorylation of GluRs. In line with this, it has been shown that A_2A_Rs stimulation leads to the potentiation of glutamatergic currents in hippocampal neurons and to GluR1 phosphorylation (Dias et al., 2012). It has to be considered that both A_1_R and A_3_R are coupled to Gi/Go proteins, and that their activation leads to a decrease in the level of cAMP (Palmer et al., 1995; Lorenzen et al., 1998). It can be thus hypothesized that the concomitant block of A_1_R and A_3_R will consistently increase the level of cAMP, possibly leading to a bigger effect respect to the one observed with a single receptor antagonist.

The effect of ARs stimulation on the amplitude of AMPA-evoked currents is observed both in hippocampal cultures and in acute slices. It can be speculated that the modulation of agonist affinity may have relevance only on extrasynaptic GluRs, physiologically activated by low concentration of glutamate, as it is has been shown for adenosine modulation of GABA receptors (Rombo et al., 2014). Indeed, our results show that the ARs stimulation by NECA, in particular A_3_R activation, also affects glutamatergic synaptic transmission in hippocampal slices, where GluRs are activated by high transmitter concentration (Clements, 1996), depressing EPSCs amplitude. Moreover, EPSCs depression is not associated to a change in the paired pulse ratio, indicating a postsynaptic mechanism (Dobrunz and Stevens, 1997).

It should be noted that EPSCs depression induced by NECA shows a peculiar pharmacology, likely reflecting a preferential action of the agonist on postsynaptic A_3_R, due to the higher NECA affinity for A_3_R compared to other ARs (Müller, 2003; Müller and Jacobson, 2011; Dal Ben et al., 2014). Indeed, in our experimental conditions, extracellular adenosine might activate or desensitize presynaptic A_1_R. Thus, in addition to the well known A_1_R presynaptic modulation (Sebastião et al., 1990; Sebastião and Ribeiro, 2000, 2009; Boison, 2007), the ARs stimulation may depress EPSCs by an A_3_R-dependent postsynaptic mechanism. The involvement of A_3_R in the glutamatergic synaptic modulation has been previously reported (Brand et al., 2001; Costenla et al., 2001; Piccinin et al., 2010), particularly in hypoxic conditions (Hentschel et al., 2003; Pugliese et al., 2007), when the adenosine extracellular level is highly increased (Fredholm, 2007).

While interpreting the pharmacological results, it should be considered that basal adenosine may affect ARs to different extent; due to their desensitization properties (Klaasse et al., 2008) and that phenomena of re-sensitization may occur (Kompa and Summers, 1999). In this respect, we consider convincing the consistency of data obtained with pharmacological and knock out approaches.

Results from this study clearly point to a role of basal adenosine in glutamatergic modulation; our data from hippocampal cultures show that removing different ARs, either by pharmacological or genetic approaches, causes changes in the functional properties of AMPA receptors (current amplitude and affinity for the agonist), pointing to tonic receptor modulation by ambient adenosine. Indeed, the AMPA current reduction induced by 2-Cl-IB-MECA was more pronounced in the presence of ADA (see Piccinin et al., 2010), suggesting that basal adenosine might occlude the effect of exogenous agonist application.

It could be speculated that ADA might modulate *per se* AMPA receptor activity, however, although it has been hypothesized the ability of ADA to act independently from its enzymatic activity (Franco et al., 1997; Yamashita et al., 2012), no data have been reported concerning the effect of extracellular ADA on the modulation of neurotransmitter receptors.

It should be noted that purines are released by brain cells through different mechanisms (Latini and Pedata, 2001) and that adenosine concentration may rise to the nanomolar range in hippocampal slices (Dale et al., 2000). It can be speculated that this situation does not reflect the physiological status, representing one of the effects connected to slice or culture unnatural conditions (zur Nedden et al., 2011). On the other hand, high adenosine level may be present in restricted sites during high level of activity or in pathological conditions (Ribeiro et al., 2003; Wall and Dale, 2007).

Our results show that adenosine may influence the glutamatergic neurotransmission through several independent mechanisms; in particular we show that the modulation of AMPA receptor by A_1_R (Lauro et al., 2008) is synergic with that of A_3_R, counteracting A_2A_R-dependent AMPA receptor potentiation (see also Dias et al., 2012). A_1_R, A_2A_R and A_3_R are widely expressed at hippocampal synapses, both pre- and post-synaptically (Lopes et al., 2003; Rebola et al., 2005). ARs are also expressed on glial cells (Burnstock et al., 2011), however, in our conditions, the exclusive expression of A_3_R on glial cells was not sufficient to rescue the AMPA receptor modulation in A_3_R KO cultures, highlighting the role of neuronal A_3_R in A_3_R-dependent modulation.

It should be emphasized that the relevance and even the sign of adenosine modulation of synaptic transmission and plasticity (Scianni et al., 2013) will strongly depend on the level of expression and the regional distribution of different AR subtypes (Dixon et al., 1996; Fredholm and Svenningsson, 2003; Fukumitsu et al., 2005; Haeusler et al., 2015). We speculate that this pathway of synaptic modulation could be suitable for the independent tuning of single synapses (West and Grace, 2002). In addition, it has been proposed that due to time and space constraints, the source of adenosine may determine the AR subtype to be activated: adenosine originating from ATP released by astrocytes seems to target excitatory A_2A_R (Cunha et al., 1994), while released adenosine seems to prefer A_1_R (Lovatt et al., 2012).

Since A_3_R is known to have lower affinity for adenosine (Müller and Jacobson, 2011, but see Fredholm et al., 2001b) compared to A_1_R and A_2A_R, its activity may be relevant especially after acute brain insults, such as in ischemia and traumatic brain injury, when the extracellular level of adenosine dramatically increases (Fredholm, 2007). In particular, the A_3_R activation can contribute to reduce over-excitability in order to counteract brain damage.

## Conclusion

We show that adenosine, an ubiquitous brain mediator, may tune synaptic transmission through multiple mechanisms depending on the AR subtype expression. Such mechanisms of regulation are potentially highly relevant in the fine regulation of single synapses in relation to the level of synaptic activity or other conditions leading to the local increase of adenosine concentration.

## Conflict of Interest Statement

The authors declare that the research was conducted in the absence of any commercial or financial relationships that could be construed as a potential conflict of interest.
