# Supplementary material for: Basal adenosine modulates the functional properties of AMPA receptors in mouse hippocampal neurons through the activation of A1R A2AR and A3R
Source: Front Cell Neurosci. 2015 Oct 12;9:409. doi: 10.3389/fncel.2015.00409 (PMC4601258; doi:10.3389/fncel.2015.00409)
Supplement: Supplementary file 1 [file Image_1.PDF]

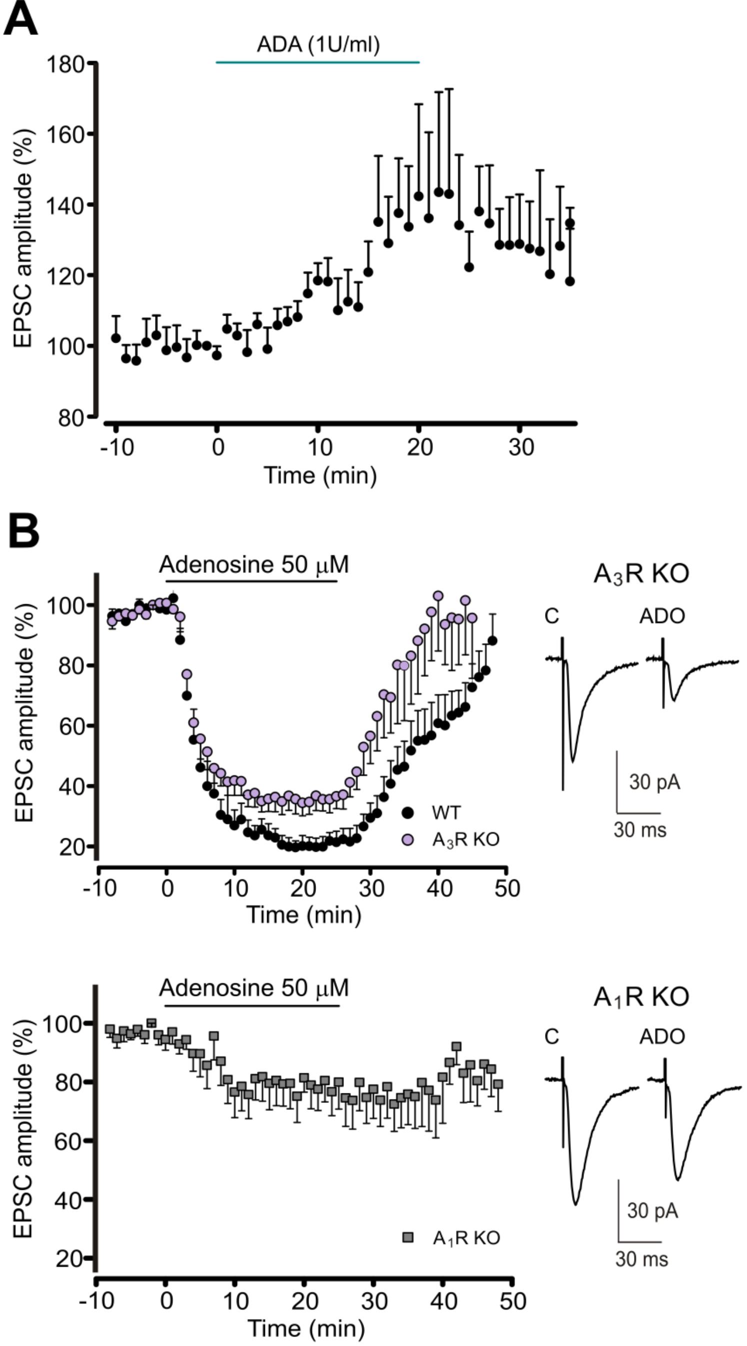

Di Angelantonio et al.
Supplementary Figure

A.Basal activation of Ars modulates EPSC in hippocampal slices. Time course of EPSC potentiation induced in CA1 pyramidal neurons by treatment with Adenosine deaminase (ADA, 1U/ml ,n = 7).

B.Modulation of EPSC amplitude by adenosine depends both on A_1R and A_3R. Time course of the effect of adenosine (50 µM) on EPSC amplitude in slices from WT (top, black circles; n = 11) and A_3RKO (top, pink circles; n = 12) or A_1RKO (bottom, grey squares; n = 10) mice.
